# Staphylococcus aureus Exploits the Host Apoptotic Pathway To Persist during Infection

**DOI:** 10.1128/mBio.02270-19

**Published:** 2019-11-12

**Authors:** Volker Winstel, Olaf Schneewind, Dominique Missiakas

**Affiliations:** a Department of Microbiology, University of Chicago, Chicago, Illinois, USA; University of Rochester

**Keywords:** *Staphylococcus aureus*, adenosine synthase A (AdsA), caspase-3, deoxyadenosine, neutrophil extracellular traps (NETs)

## Abstract

Staphylococcus aureus is a deadly pathogen that causes fatal diseases in humans. During infection, S. aureus secretes nuclease (Nuc) and adenosine synthase A (AdsA) to generate cytotoxic deoxyadenosine (dAdo) from neutrophil extracellular traps which triggers noninflammatory apoptosis in macrophages. In this manner, replicating staphylococci escape phagocytic killing without alerting the immune system. Here, we show that mice lacking caspase-3 in immune cells exhibit increased resistance toward S. aureus. Caspase-3-deficient macrophages are resistant to staphylococcal dAdo and gain access to abscess lesions to promote bacterial clearance in infected animals. We identify specific single nucleotide polymorphisms in *CASP3* as candidate human resistance alleles that protect macrophages from S. aureus-derived dAdo, raising the possibility that the allelic repertoire of caspase-3 may contribute to the outcome of S. aureus infections in humans.

## Abstract (precis)

Caspase-3 controls the apoptotic pathway, a form of programmed cell death designed to be immunologically silent. Polymorphisms leading to reduced caspase-3 activity are associated with variable effects on tumorigenesis and yet arise frequently. Staphylococcus aureus is a human commensal and a frequent cause of soft tissue and bloodstream infections. Successful commensalism and virulence can be explained by the secretion of a plethora of immune evasion factors. One such factor, AdsA, destroys phagocytic cells by exploiting the apoptotic pathway. However, human *CASP3* variants with loss-of-function alleles shield phagocytes from AdsA-mediated killing. This finding raises the possibility that some caspase-3 alleles may arise from exposure to S. aureus and other human pathogens that exploit the apoptotic pathway for infection.

## INTRODUCTION

Staphylococcus aureus, a commensal of the human skin and nares, is also an invasive pathogen causing skin and soft tissue infections, osteomyelitis, pneumonia, septic arthritis, bacteremia, and endocarditis (1, 2). Combined with antibiotic resistance, S. aureus infections are associated with high mortality rates in hospitalized patients (3, 4). Bloodstream infections with S. aureus, when not fatal, result in the seeding of abscess lesions in nearly all organs. At first, lesions appear as small areas filled with neutrophils that are attracted to invading staphylococci (5–7). Over a short period of time, however, these lesions mature to reveal a staphylococcal abscess community encased within a pseudocapsule and surrounded by layers of neutrophils and other immune cells (8, 9). This process is typically accompanied by liquefaction necrosis, i.e., the formation of pus, and the deposition of fibrin shields that protect healthy tissue from the cuff of dying neutrophils (8, 9). Thus, in spite of large numbers of immune cells, infected hosts are unable to eliminate staphylococci from abscess lesions. Lesions are slowly pushed toward organ surfaces and eventually rupture, releasing purulent exudate and staphylococci for renewed entry into the bloodstream or dissemination to new hosts (9, 10).

Although neutrophils use extracellular traps (NETs) to entangle staphylococci (11, 12), NETs are degraded by secreted staphylococcal nuclease (Nuc) and thereby fail to exert bactericidal activity (13). Nuclease digestion of NETs releases 5′- and 3′-monophosphate nucleotides that are converted by S. aureus adenosine synthase A (AdsA), a sortase-anchored surface protein, into deoxyadenosine (dAdo) (14). dAdo is toxic to macrophages and other immune cells (14, 15). In mice, S. aureus mutants lacking *adsA* exhibit diminished survival in host tissues and defects in the pathogenesis of bloodstream infections (16). AdsA-mediated dAdo production has been proposed to trigger caspase-3-induced apoptosis of mouse and human macrophages. In this model, phagocyte access to the staphylococcal abscess community, the core of staphylococcal abscess lesions, is prevented thereby promoting bacterial survival within the lesion (14). The mechanism of staphylococcal dAdo cytotoxicity was investigated using CRISPR/Cas9 mutagenesis to show that destruction of human U937 macrophages involves uptake of dAdo via the human equilibrative nucleoside transporter 1 (hENT1), dAdo conversion to dAMP by deoxycytidine kinase (DCK) and adenosine kinase (ADK), and subsequent dATP formation (17). Here, we investigate the subsequent activation of caspase-3-induced cell death upon dATP formation. We show that *CASP3*^−/−^ macrophages are resistant to AdsA-derived dAdo and that animals lacking *CASP3* expression in hematopoietic cells, including macrophages and dendritic cells, are less susceptible to S. aureus infection. We also explore how single nucleotide polymorphisms (SNPs) in human *CASP3* may protect macrophages from staphylococcal dAdo and may account for the varied susceptibility toward S. aureus disease in the human population.

## RESULTS

### Deoxyadenosine triggers caspase-3 activation in human macrophages.

**FIG 1:**
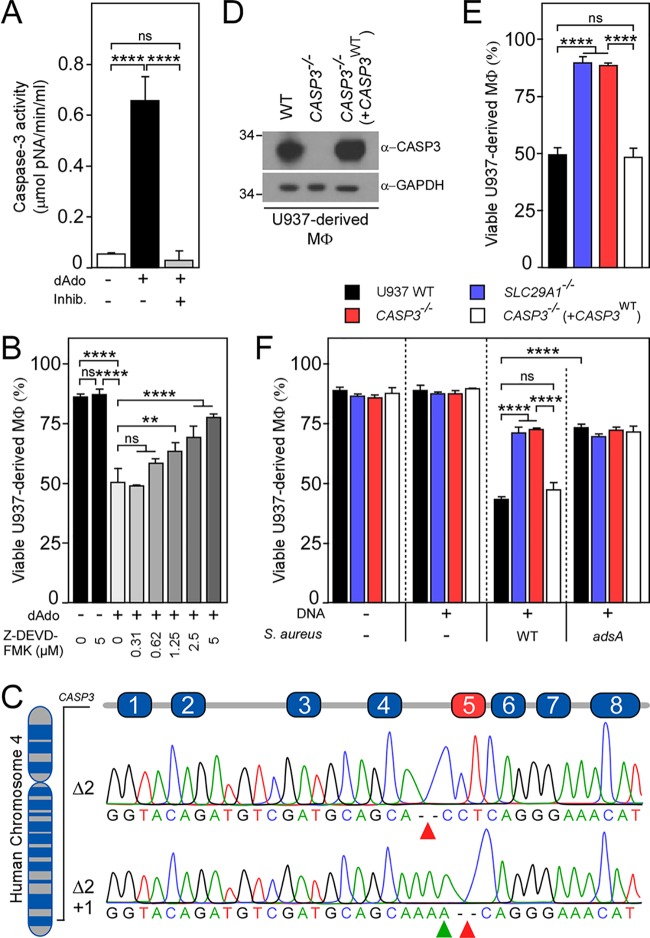
Caspase-3 is required for deoxyadenosine-induced killing of macrophages. (A) U937-derived macrophages (MΦ) were treated with (+) or without (−) dAdo, and cell lysates were analyzed for caspase-3 activity using a colorimetric assay. As controls, lysates were treated with the caspase-3 inhibitor Ac-DEVD-CHO (+ Inhib.). (B) Survival of MΦ exposed to dAdo and increasing concentrations of Z-DEVD-FMK (0 to 5 μM), an inhibitor of caspase-3. (C) Diagram illustrating the position of *CASP3* on chromosome 4 and exons 1 to 8 of *CASP3* mRNA. Sequencing results for mutated exon 5 alleles (red box) cloned from *CASP3*^−/−^ cells are shown. (D) Immunoblotting of lysates from wild-type (WT) MΦ and their *CASP3*^−/−^ and complemented *CASP3*^−/−^ variants (+*CASP3*^WT^) with caspase-3 and GAPDH-specific antibodies (α-CASP3 and α-GAPDH, respectively). GAPDH was used as a loading control. Numbers to the left of blots indicate the migration of molecular weight markers in kilodaltons. (E and F) Survival of MΦ (black bars) and their *SLC29A1*^−/−^ (blue bars), *CASP3*^−/−^ (red bars), and complemented *CASP3*^−/−^ variants (+*CASP3*^WT^, white bars) after treatment with dAdo (E) or after treatment with culture medium (RPMI 1640) that had been conditioned by incubation with either wild-type S. aureus Newman or its *adsA* mutant in the presence of host DNA, as indicated by + and – signs (F). All samples received adenosine deaminase inhibitor (50 μM dCF). Data are the mean (± standard deviation [SD]) values from three independent determinations. Statistically significant differences were analyzed with one-way analysis of variance (ANOVA) and Tukey’s multiple-comparison test; ns, not significant (*P > *0.05); **, *P < *0.01; ****, *P < *0.0001.

Earlier work revealed a correlation between staphylococcal dAdo and caspase-3 activation in macrophages surrounding abscess lesions (14). To further explore how S. aureus exploits caspase-3 activation during infection, human U937-derived macrophages were treated with 10 μM dAdo. Next, caspase-3 activity was examined in macrophage cell lysates by measuring the hydrolysis of the peptide substrate Ac-DEVD-pNA. Caspase-3 activity was undetectable in cell lysates of human macrophages left untreated (Fig. 1A). Treatment with dAdo resulted in increased caspase-3 activity in cell lysates, consistent with previous studies, and in agreement with phagocyte cell death (Fig. 1A and B) (14, 17). To account for nonspecific hydrolysis of the Ac-DEVD-pNA substrate, macrophage cell lysates were cotreated with the caspase-3 inhibitor Ac-DEVD-CHO. This treatment resulted in the loss of caspase-3 activity, confirming that dAdo provokes the specific activation of caspase-3 in human phagocytes (Fig. 1A).

### Caspase-3 is required for deoxyadenosine-mediated killing of macrophages.

To assess whether the activation of caspase-3 is responsible for phagocyte cell death, U937-derived macrophages were incubated with dAdo alone or with increasing concentrations of Z-DEVD-FMK, a membrane-permeable caspase-3 inhibitor. Preincubation with the inhibitor prevented dAdo-mediated cell death in U937-derived macrophages in a dose-dependent manner (Fig. 1B). In light of these findings, CRISPR/Cas9 mutagenesis was used to disrupt the caspase-3-encoding gene *CASP3* located on human chromosome 4 (Fig. 1C). Sanger sequencing of exon 5 targeted by the single guide RNA (sgRNA) used here confirmed the biallelic disruption of *CASP3* in U937 cells (Fig. 1C). Extracts of U937-derived *CASP3*^−/−^ macrophages (referred to as *CASP3*^−/−^ macrophages) were also analyzed by immunoblotting with caspase-3-specific antibodies, which confirmed that caspase-3 production had been abolished (Fig. 1D). *CASP3*^−/−^ macrophages were found to be refractory to dAdo-mediated toxicity in a manner similar to *SLC29A1*^−/−^ macrophages (17) that can no longer transport dAdo into the cell (Fig. 1E). Next, a plasmid encoding an sgRNA/Cas9-resistant allele of *CASP3* under the control of the EF1α promoter was transferred into *CASP3*^−/−^ macrophages (referred to as *CASP3*^−/−^ [+*CASP3*^WT^] macrophages). This process restored caspase-3 production and dAdo susceptibility (Fig. 1D and E; see also Fig. S1 in the supplemental material). Together, these experiments demonstrate that caspase-3 contributes to dAdo-mediated killing of phagocytes and suggest that *CASP3*^−/−^ macrophages should be resistant to S. aureus-derived dAdo. To test this conjecture, cultures of S. aureus Newman (wild type [WT]) or the *adsA* variant were resuspended in chemically defined medium supplemented with thymus DNA to generate conditioned culture medium. This was achieved by centrifugation of cultures to remove bacteria, followed by filter sterilization of supernatants which were then added to U937-derived macrophages or their genetic variants. In agreement with earlier work, killing of U937-derived macrophages required both S. aureus expressing *adsA* and host DNA (Fig. 1F) (14, 17). *CASP3*^−/−^ macrophages were resistant to staphylococcal dAdo in a manner comparable to *SLC29A1*^−/−^ macrophages (Fig. 1F). Genetic complementation (*CASP3*^−/−^ [+*CASP3*^WT^]) restored susceptibility to S. aureus-derived dAdo in this assay, confirming that caspase-3 is required for dAdo-mediated killing of phagocytes (Fig. 1F).

10.1128/mBio.02270-19.1 FIG S1 Engineering of a sgRNA/Cas9-resistant *CASP3* allele. (A) Diagram illustrating the coding sequence of *CASP3* targeted by the specific sgRNA used in this study. The *CASP3*-sgRNA-specific region (gray box) and the protospacer adjacent motif (PAM) are highlighted (yellow box). (B) To avoid editing by Cas9, silent mutations were introduced resulting in a sgRNA/Cas9-resistant *CASP3* allele. Nucleotide changes that do not alter the protein sequence of caspase-3 are highlighted in red. Download FIG S1, TIF file, 2.7 MB. Copyright © 2019 Winstel et al. 2019 Winstel et al. This content is distributed under the terms of the Creative Commons Attribution 4.0 International license.

### Conditional knockout mice lacking caspase-3 exhibit diminished susceptibility toward S. aureus disease.

**FIG 2:**
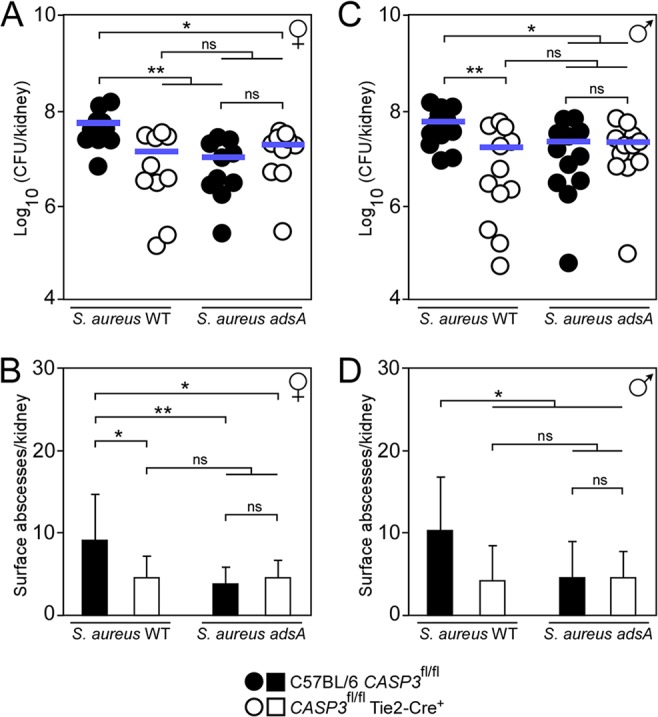
Tissue-specific deletion of caspase-3 impacts S. aureus disease pathogenesis. (A to D) Enumeration of staphylococcal loads (A and C) and visible surface abscesses (B and D) in kidneys after intravenous injection of 10^7^ CFU of wild-type S. aureus Newman or its *adsA* mutant. Data for female (♀ symbol) and male (♂ symbol) animals are displayed separately in panels A and B and in C and D, respectively. Filled (black) circles or bars indicate infection of C57BL/6 *CASP3*^fl/fl^ animals; open circles or bars indicate infection of *CASP3*^fl/fl^ Tie2-Cre^+^ mice (*n *= 10 to 12). Bacterial burden was enumerated as log_10_ CFU per kidney at 5 days postinfection. Horizontal blue bars represent the mean CFU count in each cohort (A and C) or indicate the mean (±SD) values of abscesses per kidney (B and D). Data are representative of two independent analyses. Statistically significant differences were analyzed with one-way ANOVA and Tukey’s multiple-comparison test; ns, not significant (*P > *0.05); *, *P < *0.05; **, *P < *0.01.

C57BL/6 mice with a floxed caspase-3 allele (*CASP3*^fl/fl^) have been crossed with Tie2-Cre^+^ (endothelial/hematopoietic [E+H]) mice to obtain conditional knockout animals with endothelial/hematopoietic tissue-specific deletion of caspase-3 (*CASP3*^fl/fl^ Tie2-Cre^+^ mice) (18). These animals were used to examine the contribution of caspase-3 to S. aureus pathogenesis. Control *CASP3*^fl/fl^ and *CASP3*^fl/fl^ Tie2-Cre^+^ animals were infected by intravenous inoculation of S. aureus strain Newman (10^7^ CFU). Five days postinfection, animals were euthanized. Kidneys were removed and visible abscess lesions counted before plating tissues on agar to measure bacterial loads. The analysis was conducted independently for cohorts of female and male animals. In contrast to *CASP3*^fl/fl^ female mice, bacterial loads and abscess numbers were significantly reduced in kidneys of *CASP3*^fl/fl^ Tie2-Cre^+^ female animals (Fig. 2A and B). Similarly, conditional knockout male animals were more resistant to S. aureus infection, displaying fewer abscess lesions and a significant reduction in bacterial loads in kidneys compared to *CASP3*^fl/fl^ control males (Fig. 2C and D). To test whether staphylococci manipulate host apoptosis during infection, groups of female and male animals were also challenged with S. aureus Newman lacking *adsA*. Conditional knockout animals no longer displayed increased resistance to S. aureus infection (Fig. 2). Further, infection with S. aureus *adsA* phenocopied *CASP3* loss in agreement with the notion that AdsA is required for the persistence of abscess lesions in tissues (Fig. 2) (16). In summary, these data indicate that caspase-3 contributes to S. aureus abscess formation and disease pathogenesis *in vivo* in a manner requiring staphylococcal AdsA.

### Caspase-3 affects macrophage infiltration into S. aureus abscess lesions.

**FIG 3:**
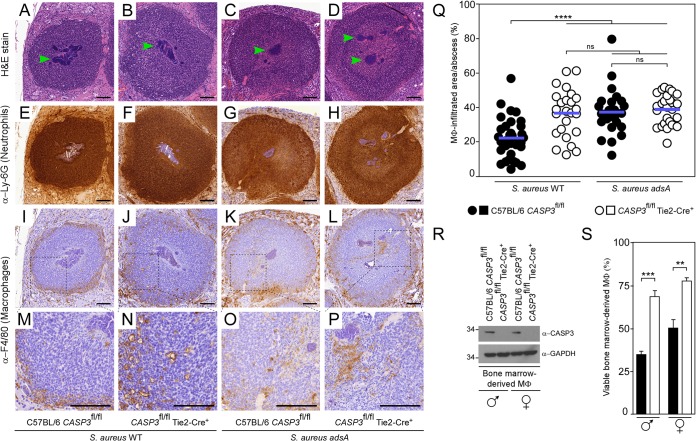
Caspase-3 activity suppresses macrophage infiltration into staphylococcal abscesses. (A to P) Immunohistochemical analysis of renal tissues isolated 5 days after intravenous injection of 10^7^ CFU of wild-type S. aureus Newman or its *adsA* mutant into C57BL/6 *CASP3*^fl/fl^ or *CASP3*^fl/fl^ Tie2-Cre^+^ mice. Thin sections were stained with hematoxylin and eosin (H&E) (A to D) or examined by immunohistochemistry with anti-Ly-6G antibodies (neutrophils) (E to H) or anti-F4/80 antibodies (macrophages) (I to L). (M to P) Magnifications of boxed area from panels I to L. Macrophages and neutrophils stain brown. Green arrows point to replicating S. aureus cells surrounded by a fibrin capsule. Black bars depict a length of 100 μm. Representative images are shown. (Q) Determination of macrophage-infiltrated areas of renal abscesses of infected C57BL/6 *CASP3*^fl/fl^ (black circles) or *CASP3*^fl/fl^ Tie2-Cre^+^ mice (open circles) by immunohistochemistry with anti-F4/80 antibodies. Macrophage-infiltrated areas were determined by calculating the total and macrophage-free (anti-F4/80-negative) abscess areas. Multiple abscesses (*n *= 25 to 35) from a cohort of 3 to 4 animals per group were analyzed. Horizontal blue bars represent mean values in each cohort. (R) Immunoblotting of lysates from bone marrow-derived macrophages (BMDM). Lysates of BMDM derived from female (♀ symbol) or male (♂ symbol) C57BL/6 *CASP3*^fl/fl^ or *CASP3*^fl/fl^ Tie2-Cre^+^ mice were probed with caspase-3 and GAPDH-specific antibodies (α-CASP3 and α-GAPDH, respectively). GAPDH was used as a loading control. Numbers to the left of blots indicate the migration of molecular weight markers in kilodaltons. (S) Survival of BMDM derived from ♀ and ♂ C57BL/6 *CASP3*^fl/fl^ (black bars) or *CASP3*^fl/fl^ Tie2-Cre^+^ mice (open bars) after treatment with dAdo and dCF (50 μM). Data are the mean (±SD) values from three independent determinations. Statistically significant differences were analyzed with one-way ANOVA and Tukey’s multiple-comparison test (Q), or by a two-tailed Student's *t* test (S); ns, not significant (*P > *0.05); **, *P < *0.01; ***, *P < *0.001; ****, *P < *0.0001.

Differences in abscess development in the kidneys of infected animals may stem from caspase-3 deficiency in hematopoietic cells. In this model, the loss of caspase-3 would protect murine phagocytes from S. aureus-derived dAdo, allowing for the infiltration of macrophages to the staphylococcal abscess community, a process otherwise restricted by staphylococcal dAdo and AdsA (14). To explore this possibility, kidneys of *CASP3*^fl/fl^ or *CASP3*^fl/fl^ Tie2-Cre^+^ animals infected with S. aureus Newman were thin-sectioned and examined using immunohistochemistry. As expected, renal abscess lesions of *CASP3*^fl/fl^ mice revealed staphylococcal abscess communities surrounded by cuffs of immune cells composed mainly of Ly-6G-positive neutrophils and mostly lacking F4/80-positive macrophages (Fig. 3A to P) (14). On the contrary, F4/80-positive macrophages were observed to be diffused throughout the neutrophil cuff of lesions from *CASP3*^fl/fl^ Tie2-Cre^+^ animals (Fig. 3A to P). To better assess macrophage infiltration, immunohistochemistry images of multiple abscesses were used to delineate the total surface area of lesion (anti-Ly-6G-positive) and surface area free of macrophages (anti-F4/80-negative). The data were used to calculate the percent area of lesions occupied by macrophages (Fig. 3Q). Wild-type *CASP3*^fl/fl^ animals restricted macrophages from accessing abscess lesions following infection with strain Newman; as expected, this restriction was lost upon infection with the *adsA* mutant (Fig. 3Q). Similarly, abscess lesions in *CASP3*^fl/fl^ Tie2-Cre^+^ mice infected with Newman contained significantly more macrophages, and macrophage recruitment no longer required *adsA* (Fig. 3Q). Thus, the *CASP3* mutation in mice phenocopies the S. aureus *adsA* mutation (Fig. 3A to Q). Next, bone marrow-derived macrophages (BMDM) were isolated from *CASP3*^fl/fl^ and *CASP3*^fl/fl^ Tie2-Cre^+^ mice. Immunoblotting confirmed the lack of caspase-3 in *CASP3*^fl/fl^ Tie2-Cre^+^ BMDM extracts (Fig. 3R). When exposed to dAdo, BMDM lacking caspase-3 exhibited increased viability compared to wild-type (*CASP3*^fl/fl^) macrophages (Fig. 3S). Together, these findings indicate that caspase-3 deficiency protects macrophages from AdsA-derived dAdo and accounts for their increased infiltration into staphylococcal abscesses.

### Human polymorphisms in *CASP3* prevent deoxyadenosine-mediated killing of macrophages.

**FIG 4:**
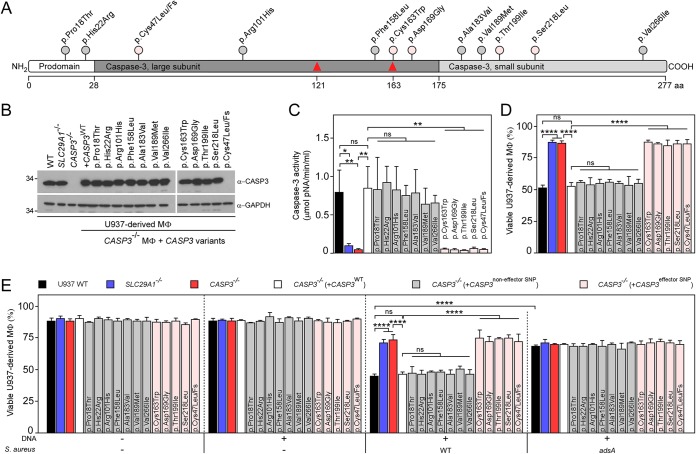
Single nucleotide polymorphisms (SNPs) in *CASP3* protect human macrophages from S. aureus-derived deoxyadenosine. (A) Caspase-3 protein lollipop plot highlighting amino acid substitutions investigated in this study. Associated SNP identifiers (IDs) are provided in Table S1. (B) Immunoblotting of lysates from wild-type (WT) U937-derived macrophages (MΦ) and their *SLC29A1*^−/−^, *CASP3*^−/−^, and complemented *CASP3*^−/−^ variants (WT and 12 different alleles indicated according to their amino acid substitution in caspase-3) using caspase-3 and GAPDH-specific antibodies (α-CASP3 and α-GAPDH, respectively). GAPDH was used as a loading control. Numbers to the left of blots indicate the migration of molecular weight markers in kilodaltons. (C) Caspase-3 activity in cell lysates of dAdo-exposed WT MΦ (black bars) and their *SLC29A1*^−/−^ (blue bars), *CASP3*^−/−^ (red bars), and *CASP3*^−/−^ variants complemented with WT (open bars) and various alleles of *CASP3* (gray or pink bars). Caspase-3 activity was measured using a colorimetric assay. (D and E) Survival of WT MΦ (black bars) and their *SLC29A1*^−/−^ (blue bars), *CASP3*^−/−^ (red bars), and *CASP3*^−/−^ variants complemented with WT (open bars) and various alleles of *CASP3* (gray or pink bars) after treatment with dAdo (D) or after treatment with culture medium (RPMI) that had been conditioned by incubation with either wild-type S. aureus Newman or its *adsA* mutant in the presence of host DNA, as indicated with + and – signs (E). (C to E) Gray indicates functional *CASP3* alleles that support caspase-3 activity, whereas pink depicts nonfunctional alleles that do not restore caspase-3 activity in *CASP3*^−/−^-derived macrophages. All samples received adenosine deaminase inhibitor (50 μM dCF). Data are the mean (±SD) from three independent determinations. Statistically significant differences were analyzed with one-way ANOVA and Tukey’s multiple-comparison test; ns, not significant (*P > *0.05); *, *P < *0.05; **, *P < *0.01; ****, *P < *0.0001.

The *CASP3* gene of humans carries many different single nucleotide polymorphisms (SNPs) (19). We wondered whether some of these SNPs may be associated with resistance to dAdo-mediated cytotoxicity and with increased resistance to S. aureus infection. To test this possibility, two publicly available databases (ExAC and dbSNP) were screened for candidate SNPs in human *CASP3*. Twelve SNPs scanning the length of caspase-3 were selected (Fig. 4A) and reconstituted into the plasmid expressing the *CASP3* sgRNA/Cas9-resistant allele. SNPs were named according to their amino acid substitution in caspase-3 (Fig. 4A). The resulting SNP-bearing constructs were transferred into *CASP3*^−/−^ macrophages, and cellular extracts examined by immunoblotting (Fig. 4B). With the exception of *CASP3*^−/−^ (+*CASP3*^p.Cys47Leu/Fs^) macrophages, which express an SNP that causes a frameshift (Fs) mutation, all *CASP3* mutant allele-expressing *CASP3*^−/−^ macrophages produced similar amounts of caspase-3 as did wild-type U937 or *CASP3*^−/−^ (+*CASP3*^WT^) macrophages (Fig. 4B). To test whether any of the selected SNPs impacted caspase-3 activity and phagocyte survival, macrophages were exposed to dAdo. Caspase-3 activity in cell lysates was monitored using the colorimetric substrate Ac-DEVD-pNA. Cell lysates of wild-type U937, *CASP3*^−/−^ (+*CASP3*^WT^), or *CASP3*^−/−^ macrophages producing caspase-3 p.Pro18Thr, p.His22Arg, p.Arg101His, p.Phe158Leu, p.Ala183Val, p.Val189Met, or p.Val266Ile variants displayed similar caspase-3 activity (Fig. 4C). Accordingly, *CASP3*^−/−^ macrophages producing caspase-3 variants that retained the ability to cleave the Ac-DEVD-pNA substrate remained susceptible to dAdo and apoptotic cell death (Fig. 4D). *CASP3*^−/−^ macrophages producing caspase-3 p.Cys163Trp, p.Asp169Gly, p.Thr199Ile, p.Ser218Leu, or p.Cys47Leu/Fs variants exhibited little to no caspase-3 activity and were protected from dAdo-mediated cytotoxicity (Fig. 4C and D). Human SNPs that rendered caspase-3 inactive also conferred resistance to S. aureus-derived dAdo in a manner requiring staphylococcal AdsA (Fig. 4E). Collectively, these data indicate that various human SNPs in *CASP3* prevent dAdo-mediated killing of phagocytes, presumably promoting macrophage survival during staphylococcal infections.

10.1128/mBio.02270-19.2 TABLE S1 Bacterial strains, cell lines, and mice used in this study. Download Table S1, DOCX file, 0.1 MB. Copyright © 2019 Winstel et al. 2019 Winstel et al. This content is distributed under the terms of the Creative Commons Attribution 4.0 International license.

## DISCUSSION

**FIG 5:**
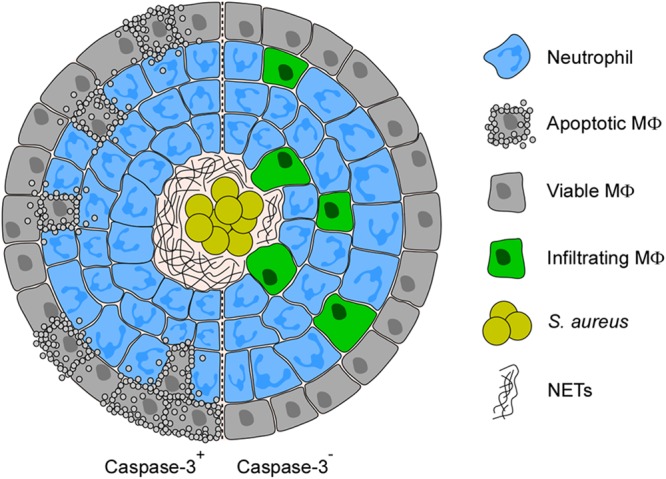
Model of macrophage exclusion from staphylococcal abscesses. Diagram illustrating the proposed role of caspase-3 during replication of S. aureus in deep-seated abscesses. Replicating S. aureus cells exploit AdsA to generate dAdo from NETs, thereby triggering caspase-3 activation and macrophage apoptosis. Caspase-3 deficiency promotes macrophage infiltration into infectious foci which affects abscess persistence and prevents the dissemination of bacteria to new foci.

The continued replication of staphylococci during infection is accompanied by the release of bacterial products (formyl peptides, lipoproteins, and peptidoglycan) and the concurrent damage of host tissues (5–7). Cellular damage triggers the release of otherwise-sequestered intracellular components, such as *N*-formylated mitochondrial peptides, nucleosomes, S100 proteins, heat shock proteins, and purines (ATP and ADP), all of which are known to potently stimulate inflammation (20–26). Nonetheless, within deep-seated abscesses, S. aureus bacteria escape phagocytic clearance to establish persistent abscess lesions (7, 27, 28). Earlier work revealed that S. aureus AdsA catalyzes the dephosphorylation of ATP, ADP, and AMP, which effectively increases the concentration of adenosine (16, 29). The activity of AdsA is reminiscent of host ectonucleoside triphosphate diphosphohydrolases and 5′-nucleotidases, which sequentially convert ATP to adenosine (30). Extracellular ATP and ADP stimulate purinergic receptors, leading to proinflammatory responses, whereas adenosine binding to cognate G protein-coupled receptors results in an anti-inflammatory response (31, 32). This mechanism allows the host to control the amplitude of inflammatory responses. Similarly, S. aureus mitigates extensive inflammation in abscess lesions and the nonending recruitment of neutrophils by producing AdsA, which increases the concentration of the anti-inflammatory mediator adenosine and reduces the concentration of proinflammatory purines. With the help of secreted staphylococcal nuclease, AdsA also generates dAdo from NETs (14). Here, we demonstrate that by doing so, staphylococci selectively kill macrophages through apoptosis, a noninflammatory cell death pathway that cannot alert the immune system. Thus, S. aureus evolved AdsA to subvert two host immune surveillance pathways and establish persistent lesions. By combining CRISPR/Cas9 mutagenesis and a renal abscess mouse model, we show that caspase-3 is required for dAdo-mediated killing of phagocytes. The immunohistochemical examination of renal tissues suggests that loss of caspase-3 renders macrophages resistant to S. aureus-derived dAdo. As a result, macrophages accumulate within abscess lesions and presumably accelerate the removal of necrotic neutrophils and remnants of NETs (Fig. 5). If so, macrophage-mediated engulfment of NETs together with entangled staphylococci probably elicits robust proinflammatory and pathogen-specific immune responses. Invading macrophages may also discharge their cellular content in order to form microbe-immobilizing macrophage extracellular traps (METs) (33–35) or directly combat replicating S. aureus in the deeper cavity of the abscess lesion, thereby supporting neutrophils in the phagocytic clearance of staphylococci (Fig. 5).

Conditional mutant animals with an endothelial/hematopoietic tissue-specific deletion of *CASP3* were used in this study, as mice lacking *CASP3* lineage dependently display neurodevelopmental abnormalities (36–38). Nonetheless, sequence analyses of human genomes reveal extensive genetic polymorphisms in *CASP3*. Some variants are associated with human cancers (39, 40), chronic periodontitis (41), and Kawasaki disease (42), raising the question of what factor may favor the maintenance of these SNPs in the human population. Here, we report that genetic polymorphisms in human *CASP3* protect macrophages from S. aureus-derived dAdo. Since humans exhibit varied susceptibility toward S. aureus infections (43, 44), we propose that recurrent staphylococcal disease and excessive generation of dAdo in abscess lesions may have contributed to the selection of some SNPs in *CASP3*. For instance, *CASP3*-inactivating SNPs, such as rs371145290 (c.653C→T, p.Ser218Leu), which predominantly occur in individuals of European ancestry, may hamper the development of abscesses as well as other staphylococcal diseases, such as endophthalmitis (45), necrotizing pneumonia (46), or mastitis (47), that have been shown to be associated with increased caspase-3 activity. Mortality rates in septic patients have also been shown to correlate with caspase-3 levels in human sera (48, 49). Thus, *CASP3* variants with reduced apoptotic activity may also influence the outcome of life-threatening sepsis. Other human pathogens synthesize dAdo, e.g., members of the genus Streptococcus (50–52), which also colonize large segments of the human population, or produce various stimuli which trigger host cell apoptosis (53). *CASP3* polymorphisms may also arise under the selective pressure of other pathogens that exploit caspase-3 activation for disease, for example, Legionnaires’ disease (54) or viral encephalitis (55, 56). Overall, interference with caspase-3 activation may determine host susceptibility toward certain infectious diseases, thereby affecting the clinical outcome of acute and recurrent infections. Thus, caspase-3, staphylococcal AdsA and its homologues represent attractive targets for new immunomodulatory therapeutic strategies to combat multidrug-resistant pathogens, including methicillin-resistant S. aureus (MRSA).

## MATERIALS AND METHODS

### Bacterial strains and growth media.

Bacterial strains were grown in Luria broth (LB; Becton, Dickinson) or tryptic soy broth (TSB; Becton, Dickinson) supplemented with the appropriate antibiotics (100 μg/ml ampicillin or 50 μg/ml kanamycin). All strains used in this study are listed in Table S1.

### Tissue culture.

U937 cells were grown in RPMI 1640 medium (Gibco) supplemented with 10% heat-inactivated fetal bovine serum (hi-FBS). HEK293FT cells were grown in Dulbecco’s modified Eagle’s medium (DMEM; Gibco) supplemented with 10% fetal bovine serum (FBS), 1 mM sodium pyruvate, 0.1 mM minimal essential medium (MEM) nonessential amino acids, 6 mM l-glutamine, and 500 μg/ml Geneticin (Gibco). Cells were grown at 37°C under 5% CO_2_. All mammalian cell lines used in this study are listed in Table S1.

### Lentivirus production.

Lentiviral particles were produced by using the ViraPower kit (Thermo Fisher), according to the manufacturer’s instructions. Lentiviral particles were harvested 48 to 72 h postinfection and concentrated by using a Lenti-X concentrator (TaKaRa), according to the manufacturer’s instructions. Lentiviral particles were suspended in DMEM, supplemented with 10% FBS and 1% bovine serum albumin, and stored at –80°C.

### Lentiviral transduction of U937 cells.

Lentiviral transduction of U937 cells was performed as described before (17). Briefly, U937 cells grown in RPMI 1640 medium supplemented with 10% hi-FBS were transduced via spinfection in the presence of 8 μg/ml Polybrene (Sigma, St. Louis, MO, USA) at a multiplicity of infection (MOI) of approximately 0.3. Viral titers were determined by transducing U937 cells (1.0 × 10^6^ cells/ml) with various volumes of lentiviral particles, along with a nonvirus-containing control via spinfection (1,000 × *g* for 2 h at room temperature). U937 cell pellets were suspended in RPMI 1640 medium containing 10% hi-FBS and incubated for 48 h at 37°C under 5% CO_2_. Cells were centrifuged, counted, and split into duplicate wells, with one well containing 2.5 μg/ml puromycin (Gibco). After 3 days, cells were counted, and the transduction efficiency was calculated as the cell count from wells containing puromycin divided by the cell count from wells without puromycin and multiplied by 100. The virus volume yielding an MOI closest to 0.3 was chosen for all experiments.

### CRISPR/Cas9 mutagenesis of U937 cells.

A LentiCRISPR v2 plasmid (57) containing a *CASP3* targeting sgRNA (ATGTCGATGCAGCAAACCTC) was purchased from GenScript (Piscataway, NJ, USA), maintained in Escherichia coli Stbl3 cells, and used to produce lentiviral particles (Table S2). CRISPR/Cas9-mediated mutagenesis was performed as described previously (17). Briefly, U937 cells were transduced by spinfection and selected with puromycin (2.5 μg/ml) for 7 days to complete gene editing. Next, single cells were isolated and clonally expanded. Genomic DNA was isolated using the DNeasy blood and tissue kit (Qiagen, Hilden, Germany). The genomic region targeted by the sgRNA and Cas9 was amplified by PCR with primers listed in Table S3 and cloned via the Zero Blunt TOPO PCR cloning kit (Thermo Fisher). Candidate plasmids from various E. coli clones were subjected to sequencing to confirm biallelic gene disruptions. All plasmids used in this study are listed in Table S2.

10.1128/mBio.02270-19.3 TABLE S2 Plasmids used in this study. Download Table S2, DOCX file, 0.1 MB. Copyright © 2019 Winstel et al. 2019 Winstel et al. This content is distributed under the terms of the Creative Commons Attribution 4.0 International license.

10.1128/mBio.02270-19.4 TABLE S3 Oligonucleotides used in this study. Download Table S3, DOCX file, 0.1 MB. Copyright © 2019 Winstel et al. 2019 Winstel et al. This content is distributed under the terms of the Creative Commons Attribution 4.0 International license.

### Analysis of human SNPs in *CASP3*.

For analysis of human SNPs and complementation studies in U937 *CASP3*^−/−^ cells, a genetically engineered *CASP3* gene refractory to sgRNA/Cas9 mutagenesis was synthesized by Integrated DNA Technologies, Inc. (Coralville, IA, USA) without changing the amino acid sequence (Fig. S1). The sgRNA-resistant *CASP3* gene was amplified via PCR and subcloned into pLVX-IRES-Neo (TaKaRa) at the XhoI and BamHI sites using primers listed in Table S3. The resulting pLVX-*CASP3*-IRES-Neo plasmid was further modified to replace the endogenous cytomegalovirus (CMV) promoter with the EF1α promoter amplified from pEF1/V5-His B (Thermo Fisher) via PCR, using the primers listed in Table S3. The new plasmid (pLVX-EF1α-*CASP3*-IRES-Neo) was maintained in E. coli Stbl3 cells and used for complementation studies. The plasmid was also used as a template to introduce various human SNPs in *CASP3* by site-directed mutagenesis using the primers listed in Table S3. The resulting plasmids were transferred into U937 *CASP3*^−/−^ cells by lentivirus-based transduction. Cells were selected with 500 μg/ml Geneticin (Gibco). Two databases, ExAC (http://exac.broadinstitute.org/) and dbSNP (https://www.ncbi.nlm.nih.gov/snp/), were screened for candidate SNPs in human *CASP3*.

### Isolation of murine bone marrow-derived macrophages.

To isolate murine BMDM, the mice were euthanized. Subsequently, the femur and tibia were removed, sterilized with 70% ethanol, and washed with sterile phosphate-buffered saline (PBS). The ends of the bones were removed to flush out the bone marrow with RPMI 1640 containing penicillin-streptomycin. Next, the bone marrow was resuspended and passed through a nylon filter (BD; 40 μm) to remove debris and unwanted tissue. Cells were centrifuged for 10 min at 200 × *g*. The cell pellet was resuspended in 3 ml red blood cell (RBC) lysis buffer (BioLegend). RBCs were lysed for 5 min at room temperature (RT), according to the manufacturer’s instructions. Cells were separated by centrifugation (10 min, RT, 200 × *g*). RBC-free cell pellets were resuspended in RPMI 1640 without penicillin-streptomycin, and cells were enumerated by using a hemocytometer. Cells were adjusted to 3.0 × 10^5^ cells/ml in BMDM medium (RPMI 1640 supplemented with 20% FBS, 1 mM pyruvate, 2 mM glutamine, 0.55 mM β-mercaptoethanol, and 10% filter-sterilized supernatant from macrophage colony-stimulating factor [CSF]-transfected 3T3-CSF cells) and seeded into 150-mm bacteriological dishes. At 3 days postextraction, cells were incubated with an additional 30 ml of BMDM medium which was entirely replaced on day 6 postextraction. BMDM were used at days 7 to 9 postextraction.

### Cytotoxicity assays.

dAdo-mediated cytotoxicity was analyzed as described elsewhere (14, 17). Briefly, 4.0 × 10^5^ U937 cells per well were seeded in a 24-well plate and incubated for 48 h at 37°C under 5% CO_2_ in RPMI 1640 medium supplemented with 10% hi-FBS and 160 nM phorbol 12-myristate 13-acetate (PMA). U937-derived macrophages were washed once and further incubated in growth medium (RPMI 1640 containing 10% hi-FBS) lacking PMA for 24 h. Alternatively, 3.5 × 10^5^ BMDM per well (obtained from *CASP3*^fl/fl^ or *CASP3*^fl/fl^ Tie2-Cre^+^ mice) were seeded in a 24-well plate and incubated for 24 h at 37°C under 5% CO_2_ in BMDM medium. U937-derived macrophages or BMDM were washed again, and media were replaced by growth or BMDM medium containing 50 μM pentostatin (2′-deoxycoformycin [dCF]) and 10 μM dAdo, as indicated in the figure legends. Cells were further incubated (U937-derived macrophages for 24 h and BMDM for 72 h) and detached using either trypsin-EDTA solution (U937-derived macrophages) or 1× PBS containing 1 mM EDTA (BMDM). Where indicated, a small-molecule inhibitor of caspase-3 (Z-DEVD-FMK; R&D Systems) was added 1 h prior to dAdo treatment. Dead cells were stained with trypan blue and counted by using a microscope to calculate killing efficiency. Cytotoxicity of S. aureus-derived dAdo was analyzed as described earlier, with minor modifications (14, 17). In brief, wild-type S. aureus Newman or *adsA* mutant cells were grown overnight in TSB, diluted 1:100 in RPMI 1640 medium, and grown at 37°C to 5.0 × 10^7^ CFU/ml. Next, 6.0 × 10^7^ CFU were incubated in RPMI 1640 containing 28 μg/ml thymus DNA (Sigma) for 3 h at 37°C. Controls lacked bacteria or thymus DNA or included the S. aureus *adsA* mutant that cannot generate dAdo (14). Bacteria were removed by centrifugation, and the resulting filter-sterilized culture supernatants were incubated with 4.0 × 10^5^ U937-derived macrophages (24-well plate) in the presence of 50 μM dCF. Cells were incubated for 18 h at 37°C under 5% CO_2_. Cells were detached using trypsin-EDTA solution, and killing efficiency was quantified with trypan blue staining.

### Immunoblotting.

U937-derived macrophages or BMDM were detached using trypsin-EDTA solution (U937) or 1× PBS containing 1 mM EDTA (BMDM), washed twice in ice-cold 1× PBS, and lysed for 20 min in ice-cold lysis buffer (50 mM HEPES [pH 7.4], 5 mM 3-[(3-cholamidopropyl)-dimethylammonio]-1-propanesulfonate [CHAPS], 5 mM dithiothreitol [DTT]). During this procedure, cells were kept on ice. Cell lysates were centrifuged for 10 min at 18,000 × *g* and 4°C. Supernatants were mixed with sodium dodecyl sulfate-polyacrylamide gel (SDS-PAGE) loading buffer and boiled at 95°C for 10 min. Proteins were separated on a 12% SDS-PAGE gel and transferred onto polyvinylidene difluoride (PVDF) membranes for immunoblot analysis with the following rabbit primary antibodies: anti-caspase-3 (anti-CASP3; for U937, antibody ab32351, and for BMDM, antibody ab13847, both from Abcam) and anti-GAPDH (loading control, PA1-987; Thermo Fisher; GAPDH, glyceraldehyde-3-phosphate dehydrogenase). Immunoreactive signals were revealed with a secondary antibody conjugated to horseradish peroxidase (Cell Signaling, Danvers, MA, USA); horseradish peroxidase activity was detected with enhanced chemiluminescent (ECL) substrate.

### Analysis of caspase-3 activity.

Caspase-3 activity was determined using the colorimetric caspase-3 detection kit (Sigma). Briefly, U937-derived macrophages were incubated in growth medium for 24 h at 37°C under 5% CO_2_ with dCF (50 μM) and dAdo (10 μM). Cells (1.0 × 10^7^), washed twice in ice-cold 1× PBS, and lysed on ice for 20 min in lysis buffer (Sigma kit). Lysates were centrifuged (18,000 × *g* for 10 min, 4°C) and supernatants incubated with the acetyl-DEVD-pNA substrate of caspase-3, according to the manufacturer’s instructions. The caspase-3 inhibitor Ac-DEVD-CHO was used in control experiments (Sigma kit). Caspase-3 activity was measured in micromoles pNA released per minute per milliliter of cell lysate.

### Animal experiments.

All animal protocols were reviewed, approved, and performed under regulatory supervision of the University of Chicago’s Institutional Biosafety Committee and Institutional Animal Care and Use Committee. *CASP3*^fl/fl^ or *CASP3*^fl/fl^ Tie2-Cre^+^ mice (C57BL/6 genetic background) (18) were obtained from Richard Flavell (Yale University, New Haven, CT) and Anthony Rongvaux (Fred Hutchinson Cancer Research Center, Seattle, WA). Mice were bred in a barrier facility at the University of Chicago. Prior to use, all animals were genotyped via PCR using the primers listed in Table S3, as described before (18). For disease studies, overnight cultures of wild-type S. aureus Newman or its *adsA* variant were diluted 1:100 in TSB and grown to an optical density at 600 nm of 0.5. Staphylococci were separated by centrifugation (10 min, RT, 8,000 × *g*), washed twice in sterile PBS, and adjusted to 10^8^ CFU/ml. Mice were anesthetized by intraperitoneal injection of 80 to 120 mg ketamine and 3 to 6 mg xylazine per kilogram of body weight. One hundred microliters of bacterial suspension (10^7^ CFU) was administered intravenously via retro-orbital injection into 6- to 8-week-old and sex-matched *CASP3*^fl/fl^ or *CASP3*^fl/fl^ Tie2-Cre^+^ mice. At 5 days postinfection, the mice were euthanized. Kidneys were dissected and homogenized in sterile PBS containing 0.1% Triton X-100. Serial dilutions were prepared and plated on tryptic soy agar (TSA) for enumeration of staphylococci. For histopathology and immunohistochemistry, dissected kidneys were fixed in 10% formalin (Fisher Scientific), embedded into paraffin, and thin sectioned. Thin sections of renal tissues were stained by the Human Tissue Resource Center (University of Chicago) with hematoxylin and eosin, or with anti-Ly-6G (neutrophils, ab210204; Abcam) or anti-F4/80 (macrophages; MCA497GA; AbD Serotec) antibodies and examined by microscopy.

### Histopathologic scoring.

Microscopic images of renal tissue thin sections stained with hematoxylin and eosin or with anti-Ly-6G (neutrophils) or anti-F4/80 (macrophages) antibodies were analyzed using the CaseViewer software (version 2.3). To calculate the macrophage-infiltrated area per abscess lesion, the total and macrophage-free (anti-F4/80-negative) abscess areas were determined. The macrophage-infiltrated area per abscess is given in the percentage relative to the total abscess area.

### Sequencing chromatograms and statistical analysis.

Sequencing chromatograms were generated with DNAStar version 12.0.0 (DNAStar Software, Inc., Madison WI, USA). Statistical analysis was performed with Prism version 7.04 (GraphPad Software, Inc., La Jolla, CA, USA). Statistically significant differences were calculated by using statistical methods, as indicated. *P* values of <0.05 were considered significant.
