# Supplementary figures and images for: Staphylococcus aureus Exploits the Host Apoptotic Pathway To Persist during Infection
Source: mBio. 2019 Nov 12;10(6):e02270-19. doi: 10.1128/mBio.02270-19 (PMC6851280; doi:10.1128/mBio.02270-19)

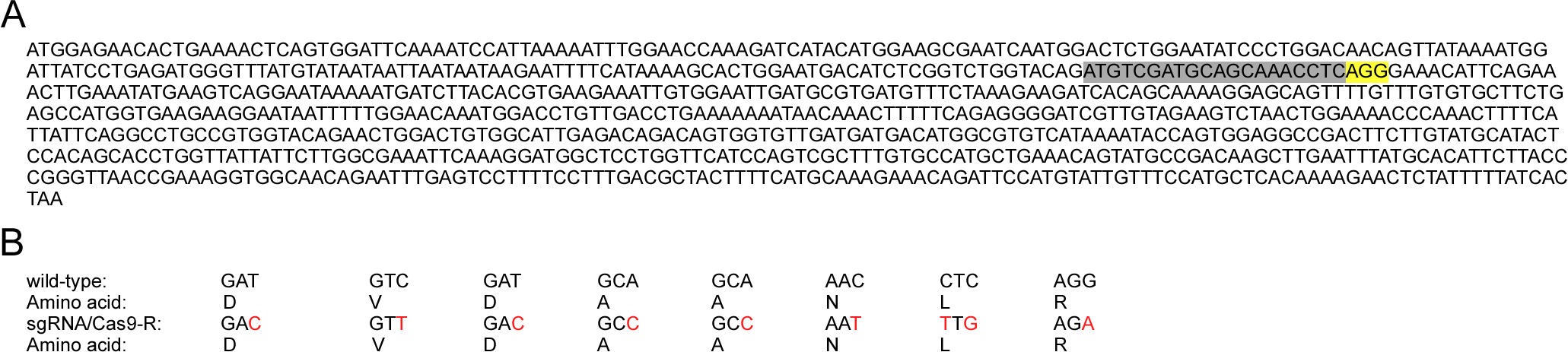

Supplement: FIG S1 [file mBio.02270-19-sf001.tif]
