# Supplementary material for: Staphylococcus aureus Exploits the Host Apoptotic Pathway To Persist during Infection
Source: mBio. 2019 Nov 12;10(6):e02270-19. doi: 10.1128/mBio.02270-19 (PMC6851280; doi:10.1128/mBio.02270-19)
Supplement: TABLE S1 [file mBio.02270-19-st001.docx]

**Table S1 – Bacterial strains, cell lines and mice used in this study**

| **Bacterial strain** | **Descriptions** | **Reference** |
| --- | --- | --- |
| *E. coli* TOP10 | One Shot® TOP10 chemically competent *E. coli,* cloning host and host strain for pUC-IDT-*CASP3* plasmid | Invitrogen |
| *E. coli* Stbl3 | Host strain used for plentiCRISPR v2 and pLVX-IRES-Neo constructs | Thermo Fisher |
| *S. aureus* Newman wildtype | *S. aureus* Newman wildtype, clinical isolate | NCTC, ID 8178 |
| *S. aureus* Newman *adsA* | *S. aureus* Newman *adsA::ermB* | Thammavongsa et al. 2013 (14) |
| **Cell line** | **Descriptions** | **Reference** |
| HEK293FT | 293FT cell line | Thermo Fisher |
| U937 wild type | U937 cell line, ATCC® CRL-1593.2™ | ATCC |
| U937 *SLC29A1^-/-^* | U937, bi-allelic deletion in *SLC29A1* | Winstel et al. 2018 (17) |
| U937 *CASP3^-/-^* | U937, bi-allelic deletion in *CASP3* | This study |
| U937 *CASP3^-/-^* (+*CASP3*^WT^) | U937 *CASP3^-/-^* with stable expression of sgRNA/Cas9-resistant caspase-3 | This study |
| U937 *CASP3^-/-^* p.Cys47Leu/Fs | U937 *CASP3^-/-^* with stable expression ofsgRNA/Cas9-resistant caspase-3 p.Cys47Leu/Fs variant; SNP ID rs1180732617 | This study |
| U937 *CASP3^-/-^* p.Cys163Trp | U937 *CASP3^-/-^* with stable expression of sgRNA/Cas9-resistant caspase-3 p.Cys163Trp variant; SNP ID rs777345631 | This study |
| U937 *CASP3^-/-^* p.Val266Ile | U937 *CASP3^-/-^* with stable expression of sgRNA/Cas9-resistant caspase-3 p.Val266Ile variant; SNP ID rs200883856 | This study |
| U937 *CASP3^-/-^* p.Asp169Gly | U937 *CASP3^-/-^* with stable expression of sgRNA/Cas9-resistant caspase-3 p.Asp169Gly variant; SNP ID rs748655755 | This study |
| U937 *CASP3^-/-^* p.His22Arg | U937 *CASP3^-/-^* with stable expression of sgRNA/Cas9-resistant caspase-3 p.His22Arg variant; SNP ID rs35578277 | This study |
| U937 *CASP3^-/-^* p.Ala183Val | U937 *CASP3^-/-^* with stable expression of sgRNA/Cas9-resistant caspase-3 p.Ala183Val variant; SNP ID rs80000647 | This study |
| U937 *CASP3^-/-^* p.Arg101His | U937 *CASP3^-/-^* with stable expression of sgRNA/Cas9-resistant caspase-3 p.Arg101His variant; SNP ID rs146285839 | This study |
| U937 *CASP3^-/-^* p.Pro18Thr | U937 *CASP3^-/-^* with stable expression of sgRNA/Cas9-resistant caspase-3 p.Pro18Thr variant; SNP ID 185559556 G/T (ExAC database) | This study |
| U937 *CASP3^-/-^* p.Ser218Leu | U937 *CASP3^-/-^* with stable expression of sgRNA/Cas9-resistant caspase-3 p.Ser218Leu variant; SNP ID rs371145290 | This study |
| U937 *CASP3^-/-^* p.Thr199Ile | U937 *CASP3^-/-^* with stable expression of sgRNA/Cas9-resistant caspase-3 p.Thr199Ile variant; SNP ID rs143138537 | This study |
| U937 *CASP3^-/-^* p.Val189Met | U937 *CASP3^-/-^* with stable expression of sgRNA/Cas9-resistant caspase-3 p.Val189Met variant; SNP ID rs1417526600 | This study |
| U937 *CASP3^-/-^* p.Phe158Leu | U937 *CASP3^-/-^* with stable expression of sgRNA/Cas9-resistant caspase-3 p.Phe158Leu variant; SNP ID rs1026750296 | This study |
| **Mouse strain** | **Descriptions** | **Reference** |
| C57BL/6 *CASP3*^fl/fl^ | C57BL/6 mice with floxed caspase-3 alleles | Rongvaux et al. 2014 (18) |
| C57BL/6 *CASP3*^fl/fl^ Tie2-Cre^+^ | C57BL/6 mice with tissue-specific (hematopoietic and endothelial) conditional deletion of caspase-3 | Rongvaux et al. 2014 (18) |
