# Supplementary material for: Staphylococcus aureus Exploits the Host Apoptotic Pathway To Persist during Infection
Source: mBio. 2019 Nov 12;10(6):e02270-19. doi: 10.1128/mBio.02270-19 (PMC6851280; doi:10.1128/mBio.02270-19)
Supplement: TABLE S2 [file mBio.02270-19-st002.docx]

**Table S2 – Plasmids used in this study**

| **Plasmid** | **Descriptions** | **Reference** |
| --- | --- | --- |
| plentiCRISPRv2 | Cas9/gRNA expression plasmid, 3^rd^ generation system | Sanjana et al. 2014 (57) |
| plentiCRISPRv2-*CASP3*-sgRNA1 | Cas9/gRNA expression plasmid containing *CASP3* targeting sgRNA sequence ATGTCGATGCAGCAAACCTC | Genscript, USA |
| pEF1/V5-His B | Mammalian expression vector containing human elongation factor 1 alpha (EF1α) promoter | Thermo Fisher |
| pLVX-IRES-Neo | Bicistronic lentiviral expression vector, expression driven by human cytomegalovirus immediate early promoter | Takara |
| pUC-IDT-*CASP3* | pUC plasmid containing sgRNA/Cas9-resistant *CASP3* gene | Integrated DNA Technologies |
| pLVX-*CASP3*-IRES-Neo | Bicistronic lentiviral vector expressing sgRNA/Cas9-resistant *CASP3* gene driven by human cytomegalovirus immediate early promoter | This study |
| pLVX-EF1α-*CASP3*-IRES-Neo | Bicistronic lentiviral vector expressing sgRNA/Cas9-resistant *CASP3* gene driven by the human elongation factor 1 alpha (EF1α) promoter | This study |
| pLVX-EF1α-*CASP3*-IRES-Neo (rs1180732617) | Bicistronic lentiviral vector expressing sgRNA/Cas9-resistant caspase-3 p.Cys47Leu/Fs variant; SNP ID rs1180732617 | This study |
| pLVX-EF1α-*CASP3*-IRES-Neo (rs777345631) | Bicistronic lentiviral vector expressing sgRNA/Cas9-resistant caspase-3 p.Cys163Trp variant; SNP ID rs777345631 | This study |
| pLVX-EF1α-*CASP3*-IRES-Neo (rs200883856) | Bicistronic lentiviral vector expressing sgRNA/Cas9-resistant caspase-3 p.Val266Ile variant; SNP ID rs200883856 | This study |
| pLVX-EF1α-*CASP3*-IRES-Neo (rs748655755) | Bicistronic lentiviral vector expressing sgRNA/Cas9-resistant caspase-3 p.Asp169Gly variant; SNP ID rs748655755 | This study |
| pLVX-EF1α-*CASP3*-IRES-Neo (rs35578277) | Bicistronic lentiviral vector expressing sgRNA/Cas9-resistant caspase-3 p.His22Arg variant; SNP ID rs35578277 | This study |
| pLVX-EF1α-*CASP3*-IRES-Neo (rs80000647) | Bicistronic lentiviral vector expressing sgRNA/Cas9-resistant caspase-3 p.Ala183Val variant; SNP ID rs80000647 | This study |
| pLVX-EF1α-*CASP3*-IRES-Neo (rs146285839) | Bicistronic lentiviral vector expressing sgRNA/Cas9-resistant caspase-3 p.Arg101His variant; SNP ID rs146285839 | This study |
| pLVX-EF1α-*CASP3*-IRES-Neo (185559556 G/T) | Bicistronic lentiviral vector expressing sgRNA/Cas9-resistant caspase-3 p.Pro18Thr variant; SNP ID 185559556 G/T (ExAc database) | This study |
| pLVX-EF1α-*CASP3*-IRES-Neo (rs371145290) | Bicistronic lentiviral vector expressing sgRNA/Cas9-resistant caspase-3 p.Ser218Leu variant; SNP ID rs371145290 | This study |
| pLVX-EF1α-*CASP3*-IRES-Neo (rs143138537) | Bicistronic lentiviral vector expressing sgRNA/Cas9-resistant caspase-3 p.Thr199Ile variant; SNP ID rs143138537 | This study |
| pLVX-EF1α-*CASP3*-IRES-Neo (rs1417526600) | Bicistronic lentiviral vector expressing sgRNA/Cas9-resistant caspase-3 p.Val189Met variant; SNP ID rs1417526600 | This study |
| pLVX-EF1α-*CASP3*-IRES-Neo (rs1026750296) | Bicistronic lentiviral vector expressing sgRNA/Cas9-resistant caspase-3 p.Phe158Leu variant; SNP ID rs1026750296 | This study |
