# Supplementary material for: Staphylococcus aureus Exploits the Host Apoptotic Pathway To Persist during Infection
Source: mBio. 2019 Nov 12;10(6):e02270-19. doi: 10.1128/mBio.02270-19 (PMC6851280; doi:10.1128/mBio.02270-19)
Supplement: TABLE S3 [file mBio.02270-19-st003.docx]

**Table S3 – Oligonucleotides used in this study**

| **Primer** | **Sequence** | **Application** | **Reference** |
| --- | --- | --- | --- |
| CASP3-floxed-A | GAGCCTTCATAGGGGTGCAA | Mouse typing | Rongvaux et al. 2014 (18) |
| CASP3-floxed-B | GGGGAGCAGAGGGAATAAAG | Mouse typing | Rongvaux et al. 2014 (18) |
| CASP3-floxed-C | CATAGAATCCCAAGCCAGGA | Mouse typing | Rongvaux et al. 2014 (18) |
| Cre-fwd | CGATGCAACGAGTGATGAGG | Mouse typing | Rongvaux et al. 2014 (18) |
| Cre-rev | CGCATAACCAGTGAAACAGC | Mouse typing | Rongvaux et al. 2014 (18) |
| CPP-typ1-up | AAACCGCTGTGAAAGACATCATTTTGGCTA | Amplification of genomic region targeted by the *CASP3* specific sgRNA | This study |
| CPP-typ1-dn | GACTAAAACTAAGAGGTCATCTTAACCTAACCT | Amplification of genomic region targeted by the *CASP3* specific sgRNA | This study |
| pUC-CASP3up | ATCCTCGAGGCCACCATGGAGAACACTGAAAACTCAGTGGATTCA | Amplification of sgRNA/Cas9-resistant *CASP3* from pUC-IDT-*CASP3* | This study |
| pUC-CASP3dn | GAGAGGATCCTTAGTGATAAAAATAGAGTTCTTTTGTGAGCATGG | Amplification of sgRNA/Cas9-resistant *CASP3* from pUC-IDT-*CASP3* | This study |
| LX-EF1-A | GGATCTATTTCCGGTGAATTCCTCGAG | Amplification of pLVX-*CASP3*-IRES-Neo without CMV promoter | This study |
| LX-EF1-B | ATCGATAAACTGGATCTCTGCTGTCCCT | Amplification of pLVX-*CASP3*-IRES-Neo without CMV promoter | This study |
| LX-EF1-C | CAGAGATCCAGTTTATCGATGCTCCGGTGCCCGTCAGTGGGCAGA | Amplification of EF1α from pEF1/V5-His B | This study |
| LX-EF1-D | AATTCACCGGAAATAGATCCTCACGACACCTGAAATGGAAGAAAAAAACTTTGAAC | Amplification of EF1α from pEF1/V5-His B | This study |
| rs1180732617-up | CCTGAGATGGGTTTATTATAATAATTAATAATAAG | Site-directed mutagenesis | This study |
| rs1180732617-dn | CTTATTATTAATTATTATAATAAACCCATCTCAGG | Site-directed mutagenesis | This study |
| rs777345631-up | CATTATTCAGGCCTGGCGTGGTACAGAACTG | Site-directed mutagenesis | This study |
| rs777345631-dn | CAGTTCTGTACCACGCCAGGCCTGAATAATG | Site-directed mutagenesis | This study |
| rs200883856-up | CAGATTCCATGTATTATTTCCATGCTCACAAAAG | Site-directed mutagenesis | This study |
| rs200883856-dn | CTTTTGTGAGCATGGAAATAATACATGGAATCTG | Site-directed mutagenesis | This study |
| rs748655755-up | GTGGTACAGAACTGGGCTGTGGCATTGAGAC | Site-directed mutagenesis | This study |
| rs748655755-dn | GTCTCAATGCCACAGCCCAGTTCTGTACCAC | Site-directed mutagenesis | This study |
| rs35578277-up | GGAACCAAAGATCATACGTGGAAGCGAATCAATG | Site-directed mutagenesis | This study |
| rs35578277-dn | CATTGATTCGCTTCCACGTATGATCTTTGGTTCC | Site-directed mutagenesis | This study |
| rs80000647-up | GTTGATGATGACATGGTGTGTCATAAAATACCAG | Site-directed mutagenesis | This study |
| rs80000647-dn | CTGGTATTTTATGACACACCATGTCATCATCAAC | Site-directed mutagenesis | This study |
| rs146285839-up | GAAATTGTGGAATTGATGCATGATGTTTCTAAAGAAG | Site-directed mutagenesis | This study |
| rs146285839-dn | CTTCTTTAGAAACATCATGCATCAATTCCACAATTTC | Site-directed mutagenesis | This study |
| 185559556-up | CCATTAAAAATTTGGAAACAAAGATCATACATGGAAG | Site-directed mutagenesis | This study |
| 185559556-dn | CTTCCATGTATGATCTTTGTTTCCAAATTTTTAATGG | Site-directed mutagenesis | This study |
| rs371145290-up | CTGGTTCATCCAGTTGCTTTGTGCCATGC | Site-directed mutagenesis | This study |
| rs371145290-dn | GCATGGCACAAAGCAACTGGATGAACCAG | Site-directed mutagenesis | This study |
| rs143138537-up | CTTGTATGCATACTCCATAGCACCTGGTTATTATTC | Site-directed mutagenesis | This study |
| rs143138537-dn | GAATAATAACCAGGTGCTATGGAGTATGCATACAAG | Site-directed mutagenesis | This study |
| rs1417526600-up | GTGTCATAAAATACCAATGGAGGCCGACTTCTTG | Site-directed mutagenesis | This study |
| rs1417526600-dn | CAAGAAGTCGGCCTCCATTGGTATTTTATGACAC | Site-directed mutagenesis | This study |
| rs1026750296-up | GGAAAACCCAAACTTCTCATTATTCAGGCCTGC | Site-directed mutagenesis | This study |
| rs1026750296-dn | GCAGGCCTGAATAATGAGAAGTTTGGGTTTTCC | Site-directed mutagenesis | This study |
